# Comprehensive transcriptomic analyses identify *KDM* genes-related subtypes with different TME infiltrates in gastric cancer

**DOI:** 10.1186/s12885-023-10923-1

**Published:** 2023-05-18

**Authors:** Haichao Zhang, Haoran Wang, Li Ye, Suyun Bao, Ruijia Zhang, Ji Che, Wenqin Luo, Cheng Yu, Wei Wang

**Affiliations:** 1 Department of Osteoporosis and Bone Disease, Research Section of Geriatric Metabolic Bone Disease, Huadong Hospital Affiliated to Fudan University, Shanghai Geriatric Institute, Shanghai, 200032 China; 2 Department of Anesthesiology, Zhongshan Hospital, Fudan University, Shanghai, 200032 China; 3 Department of Oncology, Shanghai Medical College, Fudan University, Shanghai, 200032 China; 4 Department of Anesthesiology, The Affiliated Suqian Hospital of Xuzhou Medical University, Suqian, 223800 Jiangsu Province China; 5 Gastrointestinal Surgery, Changshu No. 2 People’s Hospital, No.18, Taishan Road, Changshu, 215500 Jiangsu Province China; 6 Department of Clinical Laboratory, Lianshui People’s Hospital of Kangda College Affiliated to Nanjing Medical University, Huai’an, 223400 People’s Republic of China

**Keywords:** Gastric cancer, Histone lysine demethylases, Tumor microenvironment, Immune infiltration, Immunotherapy, Chemotherapy

## Abstract

Histone lysine demethylases (*KDM*s) have been reported in various malignances, which affect transcriptional regulation of tumor suppressor or oncogenes. However, the relationship between *KDM*s and formation of tumor microenvironment (TME) in gastric cancer (GC) remain unclear and need to be comprehensively analyzed.

In the present study, 24 KDMs were obtained and consensus molecular subtyping was performed using the "NMF" method to stratify TCGA-STAD into three clusters. The ssGSEA and CIBERSORT algorithms were employed to assess the relative infiltration levels of various cell types in the TME. The KDM_score was devised to predict patient survival outcomes and responses to both immunotherapy and chemotherapy.

Three *KDM* genes-related molecular subtypes were Figured out in GC with distinctive clinicopathological and prognostic features. Based on the robust *KDM* genes-related risk_score and nomogram, established in our work, GC patients’ clinical outcome can be well predicted. Furthermore, low *KDM* genes-related risk_score exhibited the more effective response to immunotherapy and chemotherapy.

This study characterized three *KDM* genes-related TME pattern with unique immune infiltration and prognosis by comprehensively analyses of transcriptomic profiling. Risk_score was also built to help clinicians decide personalized anticancer treatment for GC patients, including in prediction of immunotherapy and chemotherapy response for patients.

**Supplementary Information:**

The online version contains supplementary material available at 10.1186/s12885-023-10923-1.

## Introduction

GC is one of the most common malignant cancer and ranked as the fourth leading cause of cancer-related deaths all around the world [1–4]. Advanced treatments have helped improving the prognosis of GC. The 5-year survival of GC patients at stage IA and IB treated with surgery are between 60 and 80%. However, the 5-year survival of advanced stage tumor remain poor [5]. Thus, effective prognostic markers and potential therapeutic targets are needed to help clinicians select the most suitable therapy for GC patients.

*KDMs* are a family of enzymes that play a crucial role in the regulation of gene expression through the dynamic modification of histone proteins [6]. These enzymes catalyze the removal of methyl groups from lysine residues on histones, which in turn modulates chromatin structure and subsequently influences transcriptional activity. Mutations or aberrant expression of *KDMs* have been observed in various types of cancer, including leukemia, breast cancer, prostate cancer, lung cancer, and colorectal cancer, among others [7]. Some *KDMs* have been identified as oncogenes, promoting tumor growth and progression, while others have been found to act as tumor suppressors, preventing cancer development [8]. These diverse roles depend on the specific *KDM*, its target genes, and the cellular context [9]. For example, *KDM*s affect the methylation of *H3K4*, *H3K9*, *H3K27*, and *H3K36*, which can regulate the expression of tumor suppressor genes or oncogenes [10, 11]. Emerging evidences indicate *KDM*s are related to various cancers. In head and neck squamous cell carcinomas (HNSCC), *KDM1*, *KDM4*, *KDM5*, and *KDM6* proteins are regarded as the useful therapeutic targets [12]. However, few studies have comprehensively explored the role *KDM* demethylase genes in clinical outcomes of gastric cancer patients. Considering that targeting KDMs has become an attractive therapeutic strategy in cancer treatment and several small molecule inhibitors targeting KDMs, particularly those in the JmjC family, have been developed and are undergoing preclinical and clinical evaluation, there is an urgent need for research investigating the prognostic role of KDM genes in GC [13, 14]. This will facilitate the discovery of potential KDM-targeted therapies for the treatment of GC patients.

TME plays a crucial role in cancer development. Within the TME, factors such as CD8 + T cells and macrophages have been identified as important determinants of response to immunotherapy or chemotherapy [15, 16]. Alterations in the abundance of TME cells, such as CD8 + T cells, macrophages, and fibroblasts, have been found to be associated with clinical outcomes in a variety of cancers, including gastric cancer [17–19]. The correlation between TME cell infiltration and KDMs has seldom been reported in GC. This study aimed to integrate mRNA and genomic data for an in-depth analysis of KDMs, with the goal of uncovering the underlying relationship between KDM genes and GC tumorigenesis. The findings could offer novel insights into the application of various therapeutic treatments for GC patients, based on the regulation of histone demethylase KDMs.

## Materials and methods

### RNA expression dataset

In this study, we analyzed the RNA expression dataset from the Gene Expression Omnibus (GEO) database (GSE66229 [20]) and the TCGA-STAD cohort. TCGA databases were obtained from UCSC Xena (https://xenabrowser.net/datapages/), while somatic mutation data were downloaded from https://portal.gdc.cancer.gov/repository. Copy number variation information was extracted from UCSC Xena.

### Non-negative matrix factorization (NMF) algorithm

The NMF algorithm was utilized to examine molecular subtypes based on KDM genes. The NMF clustering function [21] was used to stratify the TCGA-STAD cohort into three distinct clusters, as shown in Tab. S1.

### Analyses of tumor microenvironment infiltration

CIBERSORT [22] and single-sample gene set enrichment (ssGSEA) analyses [23] were conducted to evaluate TME infiltration in patients from the TCGA-STAD and GSE66229 cohorts.

### Development *KDM* genes-related risk_score

Initially, differentially expressed genes (DEGs) from the three NMF clusters were overlapped. Through gene ontology (GO) analyses, 389 genes were identified as being related to the KDM phenotype. After combined with 24 *KDM*s, all genes were used to generate a gene model with 15 genes showed the highest frequencies of 359 (Tab. S2), and then, 15 genes were used to calculate risk_score by the Lasso Cox regression algorithm, as follows:

*KDM*_score = (0.30264* *ABCG4* expression) + (0.08650* *ACSS3* expression) + (0.1489* *CKAP4* expression) + (0.31486* *FXYD1* expression) + (0.04066* *GAMT* expression) + (-0.09317* *MAP3K10* expression) + (0.01091* *PCDHB5* expression) + (0.007583* *PIEZO2* expression) + (0.04638* *PSMG3* expression) + (0.002336* *RPS4Y1* expression) + (0.07975* *SNCG* expression) + (0.22821* *SYT6* expression) + (-0.13414* *TPGS1* expression) + (-0.02402* *XIST* expression) + (-0.07743* *KDM*4A expression).

The median value of *KDM*_score was used to divide patients in high- and low-risk groups. Kaplan–Meier (K-M) survival curve and immune analyses were based on high- and low-risk groups.

### Cell migration assays

In vitro experiments involved two human-derived gastric cancer cell lines: MKN-45 and SGC-7901. A control cell line (transfected with an empty vector) was established, along with two experimental cell lines (knockdown and overexpression groups). The knockdown group provided two stable cell lines constructed with shRNA sequences, while the overexpression group provided one stable cell line. The human GC cell line MKN-45 and SGC-7901 cell line were purchased from the National Cancer Institute (Bethesda, MD, USA). Transwell assays were performed by seeding 4 × 10^4^–8 × 10^4^ cells into the upper chamber (CLS3464, Corning Costar, Corning, NY, USA) with no FBS supplementation while the lower chamber was added 600 μL DMEM with 10% FBS. After 36–72 h of culture, migrated cells were fixed with 4% paraformaldehyde (G1101, Servicebio, Wuhan, Hubei, China), stained with Crystal Violet Staining Solution (C0121, Beyotime, Shanghai, China), and counted under a microscope. Transwell assays was repeated 3 times for each group, followed by statistical analysis. The statistical comparison was performed using a t-test, * indicating *P* value < 0.05; ** indicating *P* value < 0.01; *** indicating *P* value < 0.001.

### Cell scratch wound healing assay

Cells were seeded at a density of 1 × 10^5^ cells/well in six-well plates, with triplicate wells per condition. Once the cells had uniformly spread across the bottom of each well, three to four parallel lines were meticulously drawn in each well using sterile 10 μL pipette tips. Suspended cells were gently washed away, leaving the remaining adherent cells to be cultured in serum-free medium. After a 24-h incubation period, five random fields per well were examined under a light microscope. Images were captured and cells within these fields were manually counted. In this study, we highlighted the knockdown and overexpression groups to emphasize the tumor-promoting function of KDM5C in gastric cancer (Fig. 2E). Cell scratch wound healing assay was repeated 3 times for each group, followed by statistical analysis. The statistical comparison was performed using a t-test, * indicating *P* value < 0.05; ** indicating *P* value < 0.01; *** indicating *P* value < 0.001.

### Mouse models establishment

MKN-45 cell line was selected to construct stable cell line, including an overexpression (OE) cell line and a knock-down (KD) cell line as the experimental groups. Then, the transfection efficiency of *KDM5C* was confirmed by Western blotting and quantitative reverse transcription polymerase chain reaction (qRT-PCR) analyses. Antibody used for validation of *KDM5C* expression was purchased from Affinity (#DF13631). MKN-45-NC and MKN45-*KDM5C*-OE or KD cells (5 × 10^6^) were injected subcutaneously into the right and left hind flanks, respectively, of the BALB/c nude mice. The Volume of tumor = 1/2 × length × width^2^ was adopted to calculate the size of tumors.

### RNA Isolation and quantitative real-time polymerase chain reaction (RT-qPCR)

For our study, we used a total of 120 pairs of BLCA patient tissues from Lianshui People's Hospital of kangda college Affiliated to Nanjing Medical University. All patients provided written informed consent in accordance with the Institutional Review Boards of Lianshui People's Hospital of kangda college Affiliated to Nanjing Medical University.

, and the study was approved by the Ethical Committee of Lianshui People's Hospital of kangda college Affiliated to Nanjing Medical University.

To isolate total RNA, we used Trizol reagent (Invitrogen) on either cultured cells or fresh tissue samples. We then synthesized cDNA through reverse transcription using the Prime Script RT reagent kit (TaKaRa) and conducted quantitative RT-PCR with primers in the presence of the SYBR Green Realtime PCR Master Mix (Thermo). To calculate the relative abundance of mRNA, we normalized to ACTB mRNA.

### Statistical analyses

Analyses in this study were mainly based on R and Graphpad. The Kruskal–Wallis H test was used to show the difference among three cluster. Wilcox test was used to show the difference between two clusters. The log-rank test was used in survival analysis. * indicating *P* value < 0.05; ** indicating *P* value < 0.01; *** indicating *P* value < 0.001.

## Results

### Genetic variation of *KDM* genes in gastric cancer

**Fig. 1:**
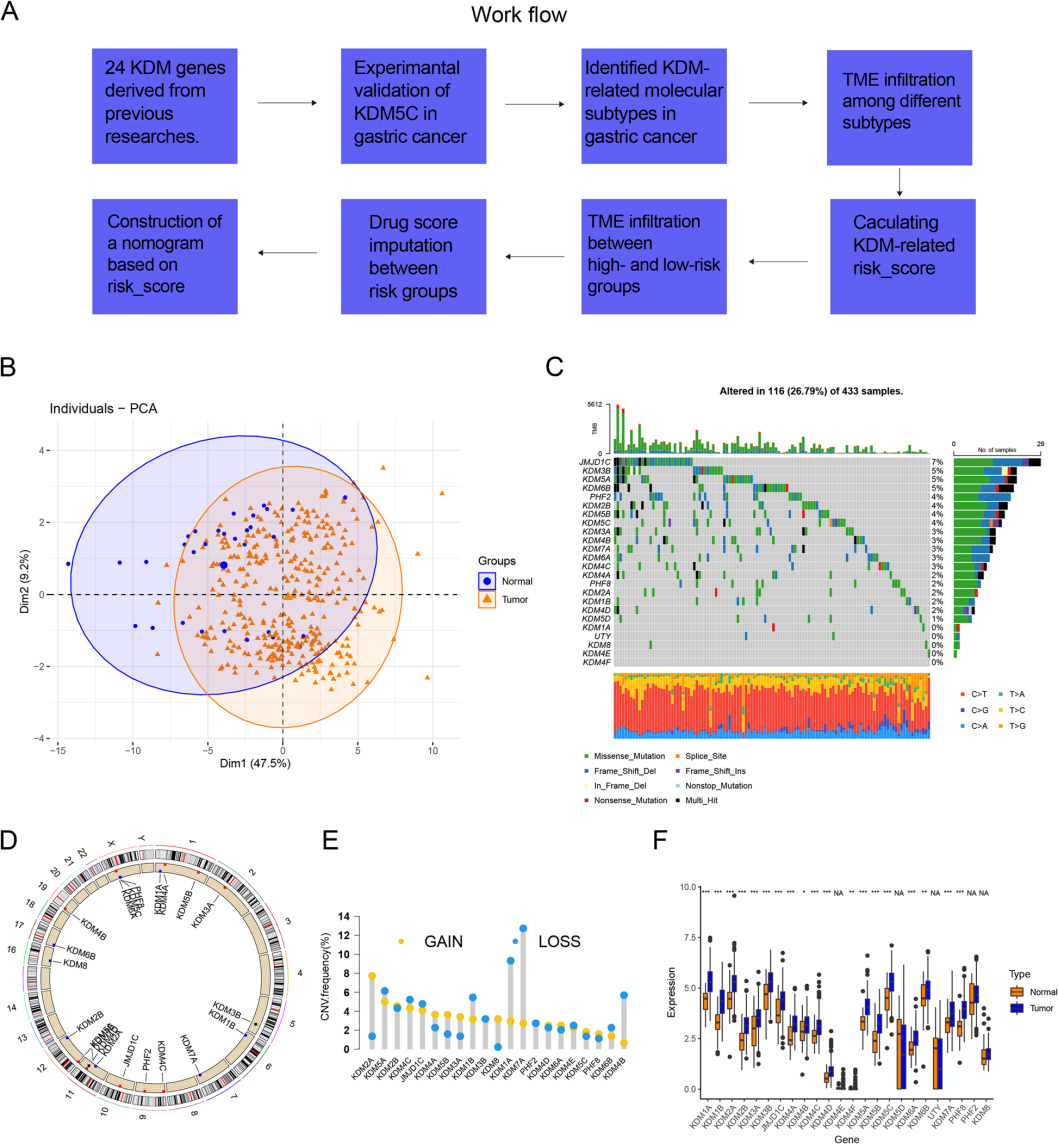
Genetic variation of *KDM* genes in gastric cancer. **A** The workflow being used in our work. **B** Using *KDM*s to discriminate tumors from normal tissue by principal component analysis (PCA). **C** Genetic alterations of 24 *KDM*s in GC tumors was demonstrated in oncoplot. Each column was each GC patient’s mutation data and mutation frequency of each gene was displayed on the right side. **D** Locations of CNV alterations in *KDM*s on 13 chromosomes. **E** 10 CRGs’ CNV diversity in GC tissues using TCGA-STAD data. **F** Boxplot shows the expression difference between normal and GC tissues in TCGA-STAD cohort. Statistical difference is identified by Wilcox test, * indicating *P* value < 0.05; ** indicating *P* value < 0.01; *** indicating *P* value < 0.001

A workflow briefly introducing our study was displayed in Fig. 1A. 24 *KDM* genes derived from previous researches [24, 25]. were included for subsequent analyses. Initially, principal component analysis (PCA) was conducted based on paired tumor-normal tissues, revealing that KDM genes could distinguish tumor tissues from normal samples in gastric cancer (Fig. 1B). Subsequently, maftools [26] was employed to screen the somatic mutations of KDM genes in the TCGA-STAD cohort. The results indicated that JMJD1C had the highest mutation rate (7%) (Fig. 1C). Copy number variations (CNV) of *KDM* genes on chromosomes were displayed in Fig. 1D. Based on CNV frequency (Fig. 1E) and RNA expression of *KDM* genes (Fig. 1F) in paired tumor-normal tissues, *KDM2A, KDM4A, KDM5B* and *KDM3A* were upregulated in tumor, consistent with their CNV amplification. These results revealed difference in the landscape of genetic alterations and expression of *KDM* genes in gastric cancer, indicating dysregulation of *KDM* genes played an important role in GC tumorigenesis.

### Experimental validation of functional phenotypes of *KDM5C* in GC

**Fig. 2:**
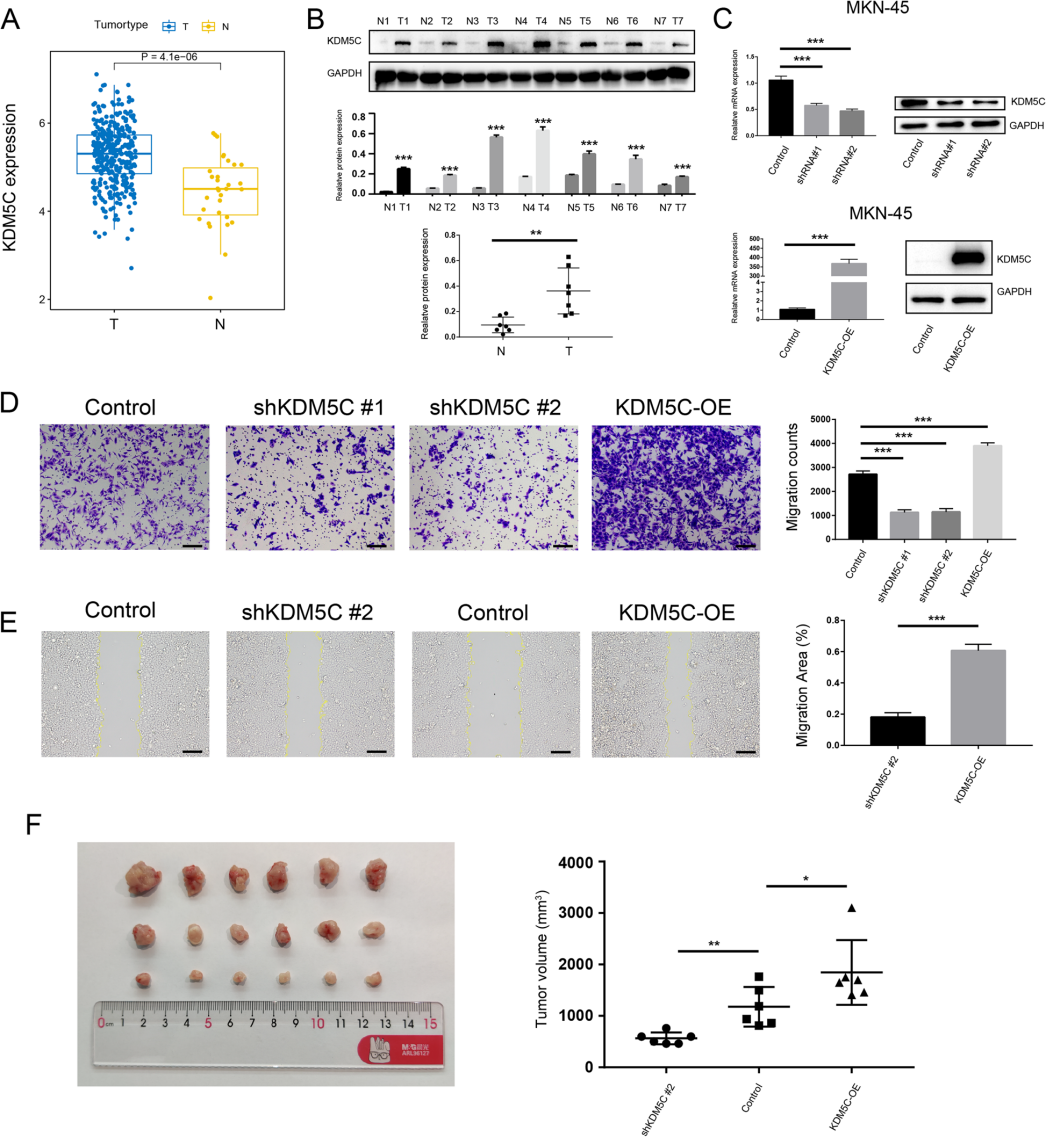
Experimental validation of functional phenotypes of *KDM5C* in GC. **A** Boxplot showed the expression of *KDM5C* between tumor and normal samples of TCGA-STAD cohort. Statistical difference is identified by t-test, * indicating *P* value < 0.05; ** indicating *P* value < 0.01; *** indicating *P* value < 0.001. **B** Western-blotting of *KDM5C* in paired normal and tumor tissues of gastric cancer. **C** Western blotting and qPCR analyses of overexpression and known-down of *KDM5C* in MKN-45 cell line. Statistical difference is identified by t-test, * indicating *P* value < 0.05; ** indicating *P* value < 0.01; *** indicating *P* value < 0.001. **D** Transwell assays of MKN-45 cell line. Statistical difference is identified by t-test, * indicating *P* value < 0.05; ** indicating *P* value < 0.01; *** indicating *P* value < 0.001. **E** Cell wound scratch assays of MKN-45 cell line. Statistical difference is identified by t-test, * indicating *P* value < 0.05; ** indicating *P* value < 0.01; *** indicating *P* value < 0.001.** F** Tumor models construction using MKN-45 cell line. Statistical difference is identified by t-test, * indicating *P* value < 0.05; ** indicating *P* value < 0.01; *** indicating *P* value < 0.001

Considering that *KDM5C* was upregulated in gastric cancer based on transcriptomic data (Fig. 2A), the higher expression of KDM5C in gastric cancer was validated using seven paired tumor-normal tissues through western blotting (Fig. 2B). In order to investigate the role of KDM5C in the metastatic potential of gastric cancer cells, KDM5C was knocked down and its expression was enhanced in the MKN-45 cell line (Fig. 2C). Results from transwell assays (Fig. 2D) and cell wound scratch assays (Fig. 2E) demonstrated that attenuated *KDM5C* expression dramatically reduced cell migration ability in vitro, while ectopic *KDM5C* expression significantly enhanced cell migration ability. Xenograft tumor assays were also conducted using the MKN-45 cell line. Overexpression of KDM5C led to accelerated xenograft tumor growth and larger tumor volumes. In contrast, knock-down of *KDM5C* resulted in an attenuated xenograft tumor growth and smaller tumor volumes (Fig. 2F). These data suggest that the tumor-promoting activity of *KDM5C* in GC.

### *KDM* genes-related molecular subtypes in GC

**Fig. 3:**
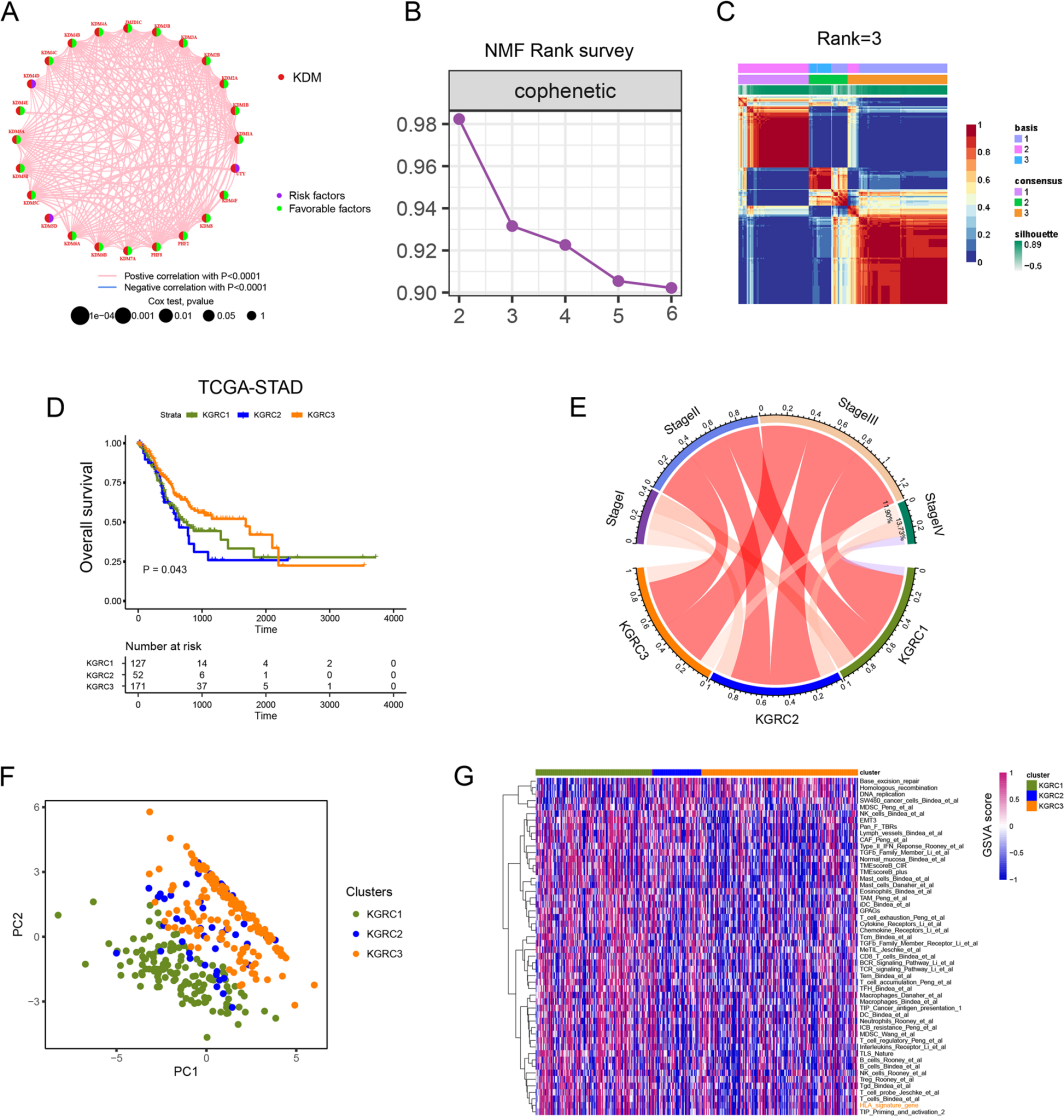
*KDM* genes-related molecular subtypes in GC. **A** Correlations and prognostic relation of 24 *KDM*s in GCs. Prognostic impact of each gene was reflected by the circle size Favorable factors for overall survival is in green, while risk factors was in purple. The line between each gene represented the correlation among CRGs. Positive correlation was in red, while negative correlation was in blue. Prognostic impact was calculated by LogRank test and correlation between genes was evaluated by Paerson analysis. **B** Plot shows the NMF rank survey and the optimal rank for cluster is 3 in TCGA-STAD cohort. **C** Consensus heatmap in TCGA-STAD cohort was shown in setting rank as 3 in NMF algorithm. **D** Kaplan–Meier survival plot for overall survival in TCGA-STAD cohort is based on 3 KGRCs sorted by NMF algorithm. *P* value was calculated by LogRank test. **E** The distribution of clinical stages (Stage I-IV) in each KGRC. **F** Principal component analysis of three KGRCs in TCGA-STAD cohort. **G** The enrichment difference of biological pathways in three KGRCs was displayed in heatmap

A network in Fig. 3A described the connections and prognostic value of *KDM* genes in GC. Next, three molecular subtypes were identified in TCGA-STAD cohort using NMF algorithm (Fig. 3B, C; Fig. S2A), as confirmed by PCA algorithm (Fig. 3F). These clusters were identified as KDM genes-related clusters (KGRCs), comprising 127 patients in KGRC1, 52 patients in KGRC2, and 171 patients in KGRC3. The survival analysis showed that KGRC2 had the worst prognosis (Fig. 3D; overall survival (OS), *P* = 0.043; log-rank test). Distribution of clinicopathological features indicated that the most of patients at stage IV were concentrated into KGRC2, supporting its corresponding prognosis patterns (Fig. 3E).

Ultimately, pathway activities were assessed using the gene set variation algorithm (GSVA) to explore the biological differences between the KGRCs (Fig. 3G; Fig. S3D). By quantification analyses (Fig. S3D), It was demonstrated that cancer-related pathways such as *Pan_F_TBR*s and *TGFb*_Family_Member_Li_et_al were predominantly enriched in KGRC2. Immune-related pathways like CD8_T_cells_Bindea_et_al and HLA_signature_gene were mainly upregulated in KGRC1. To further confirm our *KDM* genes-related classification was stable, we also included another cohort (GSE66229-ACRG) for identical analyses and obtained similar results (Fig. S3A-D). These results emphasized the significant discrepancy of biological function between different KGRCs.

### Tumor microenvironment infiltration of KGRCs

**Fig. 4:**
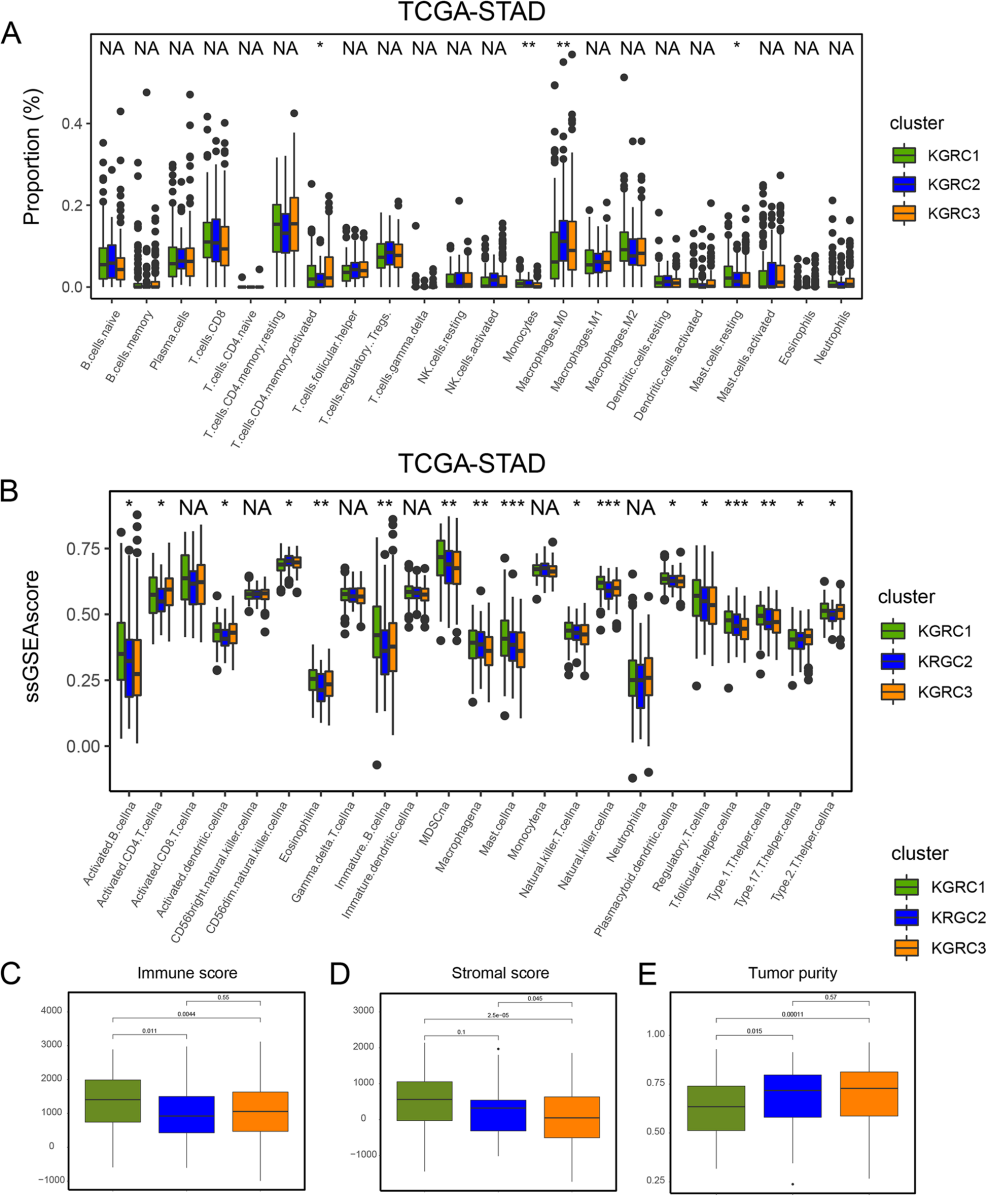
Tumor microenvironment infiltration of KGRCs. **A-B** Boxplot reflects 23 immune cells infiltration in three KGRCs using ssGSEA algorithm in TCGA-STAD cohort. Statistical difference is identified by Kruskal–Wallis H test, * indicating *P* value < 0.05; ** indicating *P* value < 0.01; *** indicating *P* value < 0.001. **C-E** ESTIMATE analyses of three KGRCs. Statistical difference is identified by Kruskal–Wallis H test, * indicating *P* value < 0.05; ** indicating *P* value < 0.01; *** indicating *P* value < 0.001

Having described the molecular differences between the three KGRCs, the TME infiltration of these clusters was next evaluated. In Fig. 4A-B, it was observed that activated CD4 + T cells were primarily enriched in KGRC1 and KGRC3, as indicated by both CIBERSORT and ssGSEA analyses. Subsequently, ESTIMATE analysis was performed in the three KGRCs, revealing that TME cells, including immune and stromal cells, were predominantly enriched in KGRC1 (Fig. 4C-D). Furthermore, KGRC1 contained the smallest proportion of tumor cells (Fig. 4E). Immune genes related to stimulation and inhibition were screened in Fig. S4A-B. Most of stimulation genes were highly expressed in KGRC1 such as *TLR4, TNFSF14,* etc. Inhibition genes such as *CD276*, *TGFB1* and *VEGFB* were highly expressed in KGRC2, in line with its poor prognosis. Therefore, the patients in KGRC1 with substantial TME cells and upregulation of immune-stimulation genes might be good candidates for immunotherapy and activated CD4^+^ T cells could be the therapeutic target to improve the prognosis of patients in KGRC1.

### Construction of *KDM*-risk score in gastric cancer

**Fig. 5:**
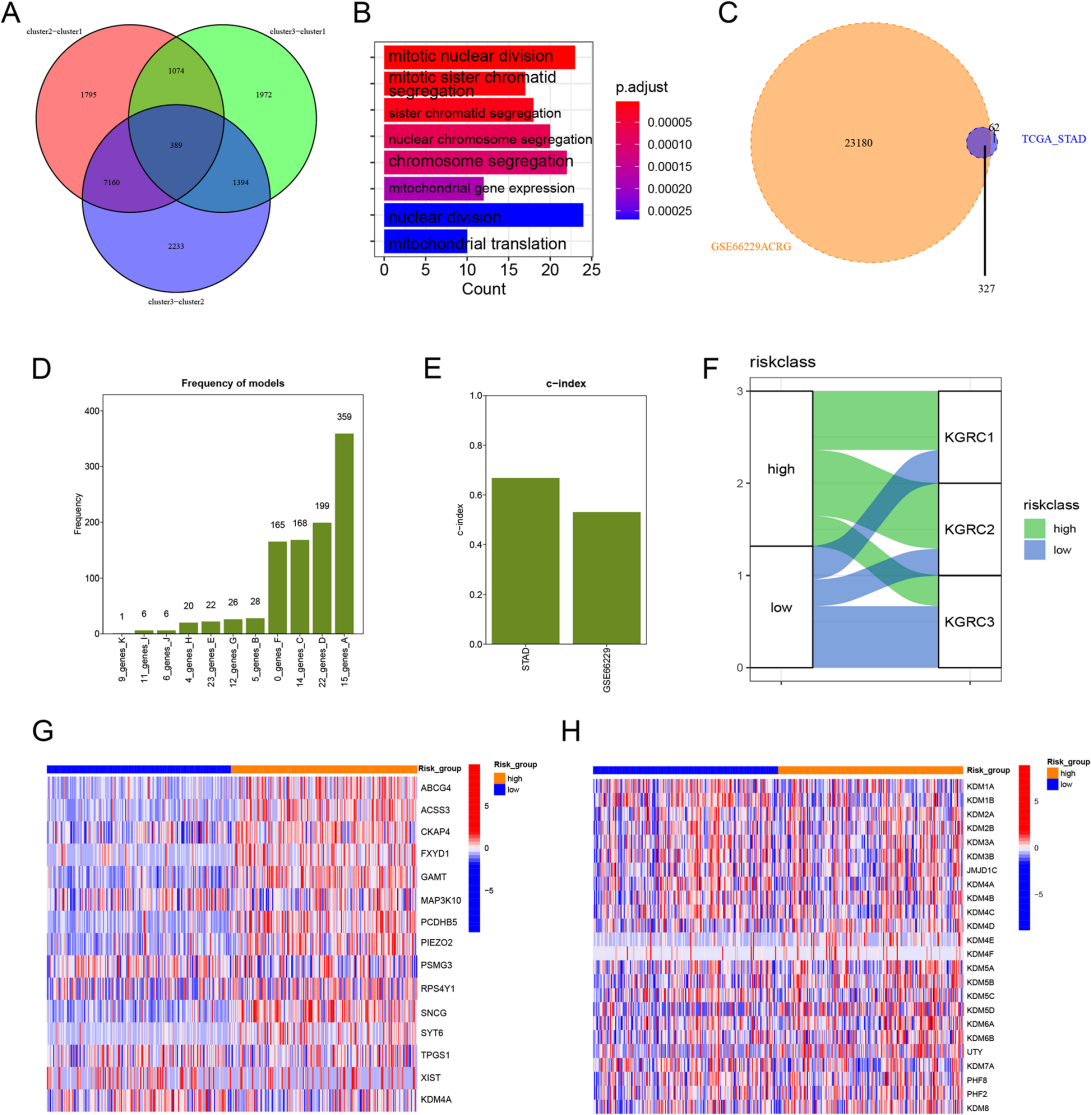
Construction of *KDM*-risk score in gastric cancer. **A** Venn plot reflected 389 *KDM* phenotype-related genes by overlapping DEGs among three KGRCs. **B** GO function enrichment of those 389 *KDM* phenotype-related DEGs. **C** Overlapping 389 genes with all genes in GSE66229. **D** Barplot showed the frequency of gene models. **E** Column plot showed the c-index of *KDM*_score in TCGA-STAD and GSE66229 cohorts. **F** Sangi plot illustrated the proportion and distribution of three KGRCs in high and low-risk group. Statistical difference is identified by Kruskal–Wallis H test, * indicating *P* value < 0.05; ** indicating *P* value < 0.01; *** indicating *P* value < 0.001. **G-H** Expression heatmap of 15 genes in building risk_score and 24 *KDM* genes was constructed in training set (TCGA-STAD) and validation cohort (GSE66229), respectively

To further comprehend the transcriptomic patterns mediated by KDM genes, a total of 389 genes were obtained by overlapping DEGs from the three KGRCs (Fig. 5A). GO analysis (Fig. 5B) revealed that these genes were associated with mitotic nuclear division and mitochondrial gene expression. These genes were identified as KDM phenotype-related signatures. In order to obtain genes for risk model construction in training and validation cohorts, the 389 genes were overlapped with all genes in a validation cohort derived from GSE66229, yielding a total of 327 genes (Fig. 5C). Subsequently, these genes and the 24 KDM genes were combined to construct the KDM-related risk_score (KDM_score). TCGA-STAD was selected as the training set, and 1000 iterations were performed as previously reported [27]. Five gene groups were obtained for screening. A group of 15 genes with the highest frequencies of 359 was ultimately selected to generate a signature for constructing the KDM_score (see methods; Fig. 5D). The c-index was used to validate the accuracy of the KDM_score in TCGA and GSE66229, as depicted in Fig. 5E. By setting the median value of the KDM_score as the threshold, the TCGA cohort was divided into high and low-risk groups. The proportion analysis showed that high-risk group was mainly clustered into previous KGRC2 with the worse prognosis (Fig. 5F). The expression levels of 15 genes used for constructing risk_score and 24 *KDM* genes between high- and low-risk groups in training cohort were shown in Fig. 5G, H.

**Fig. 6:**
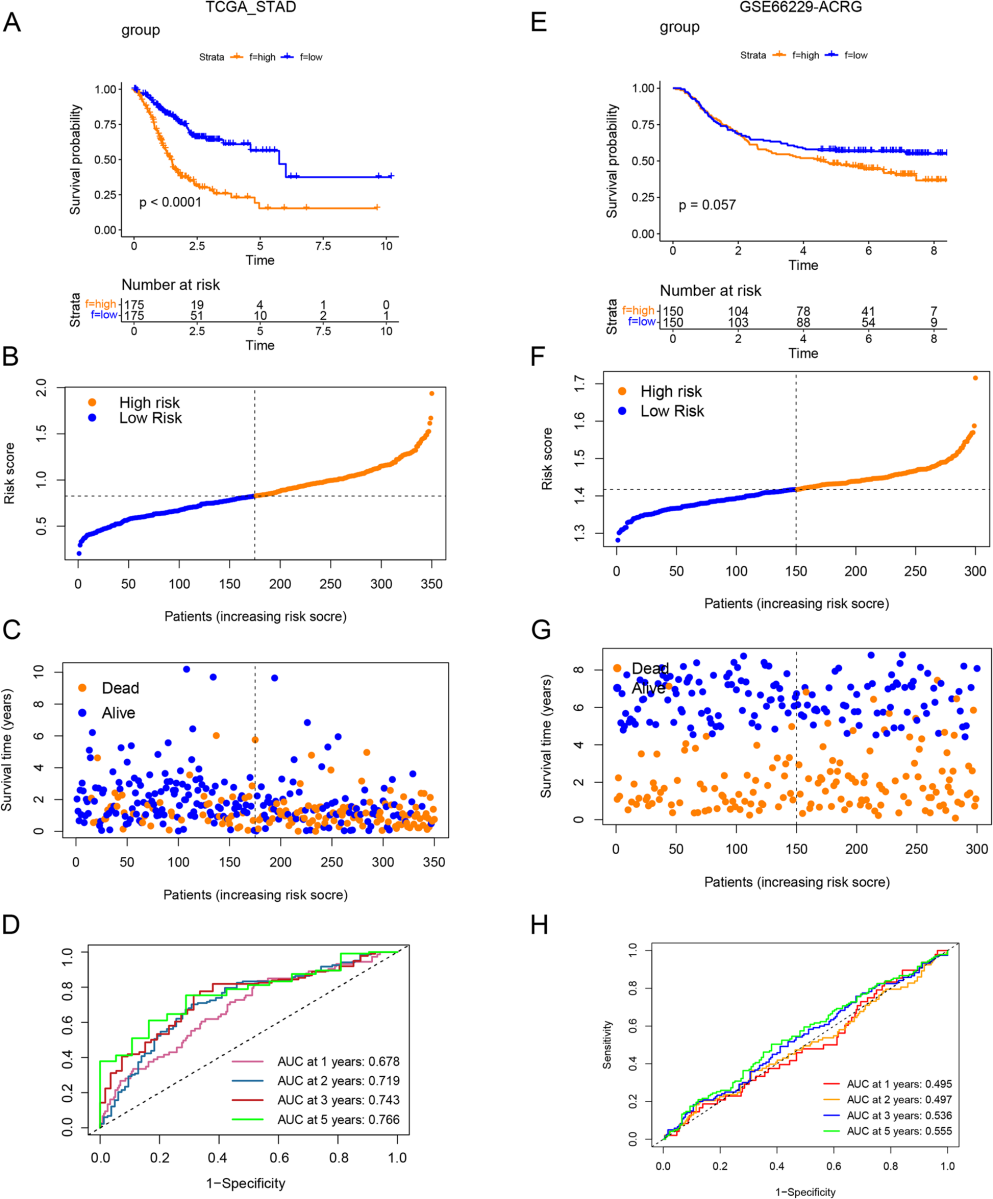
Construction of *KDM*-risk score in gastric cancer. **A, E** Kaplan–Meier survival plot in training set (TCGA-STAD) and testing set (GSE66229) is based on high and low risk group. *P* value was calculated by LogRank test. **B, C, F, G** Distribution plot reflected the relationship between dead status and risk score in training set (TCGA-STAD) and validation cohort (GSE66229), respectively. **D, H** ROC curve shows AUC values of *KDM*_score in predicting 1-, 2-, 3-, and 5-year survival of patients in validation cohort (GSE14333 and GSE37892)

Survival analyses showed that high-risk group predicted the worse prognosis in both of training (TCGA-STAD cohort) and testing cohorts (GSE66229-ACRG) (Fig. 6A, E). The distribution plot of risk scores and survival rates in all datasets showed that the high-risk groups had a higher mortality rate compared to the low-risk groups (Fig. 6B, C; Fig. 6F, G). AUC values of 1-, 2-, 3-, and 5-year survival rates in training set (TCGA-STAD) were 0.678, 0.719, 0.743, and 0.766, respectively (Fig. 6D). AUC values of 1-, 2-, 3-, and 5-year survival rates in validation cohort (GSE66229) were 0.495, 0.497, 0.536, and 0.555 (Fig. 6H). These results indicated the predictive power of *KDM*_score for survival.

### Immune-related characteristics between the high- and low-risk groups

**Fig. 7:**
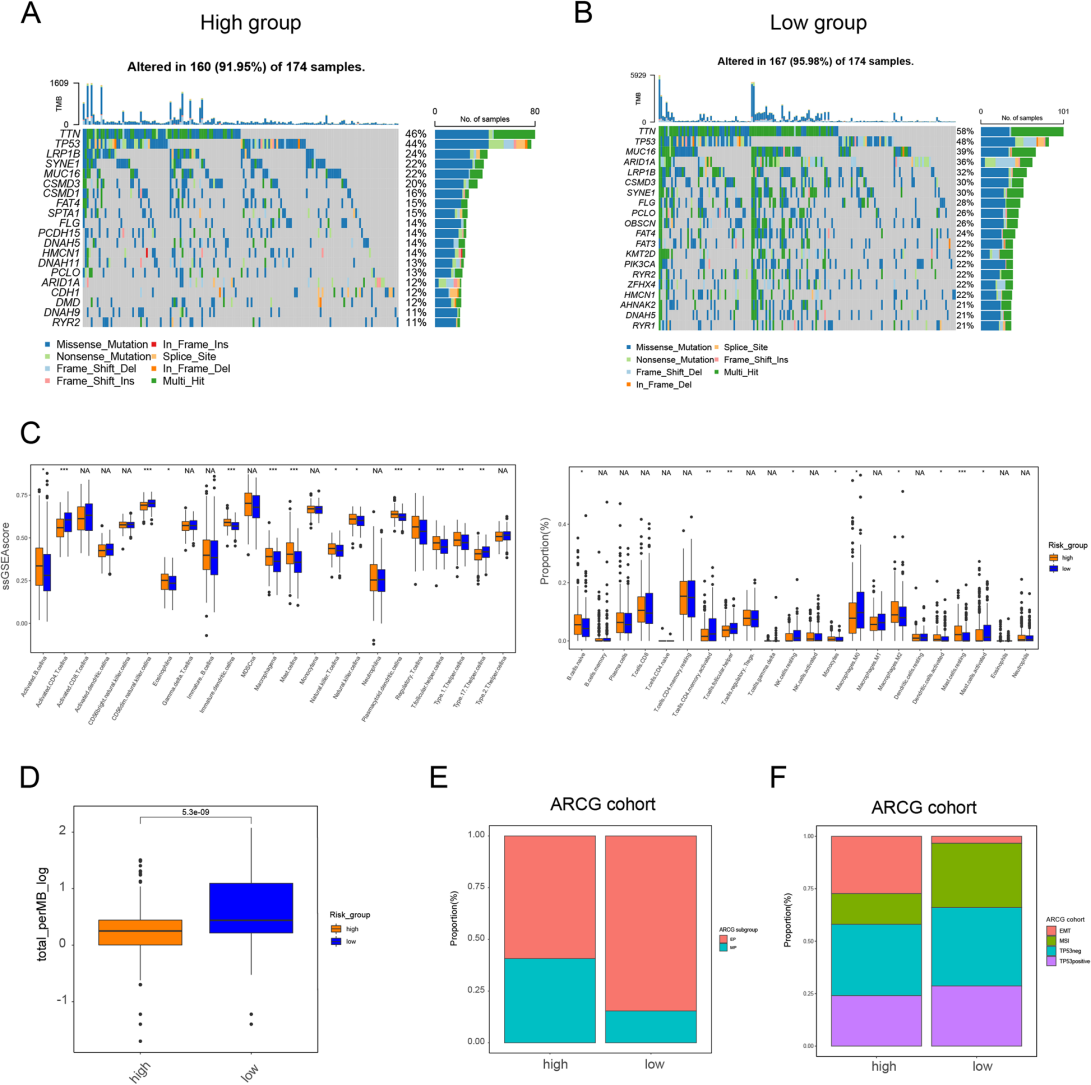
Immune-related characteristics between the high- and low-risk groups. **A, B** Oncoplots showed mutation of STAD between low and high-risk groups. **C** Boxplot reflects 23 immune cells infiltration in three KGRCs using ssGSEA algorithm in TCGA-STAD cohort. Statistical difference is identified by Kruskal–Wallis H test, * indicating *P* value < 0.05; ** indicating *P* value < 0.01; *** indicating *P* value < 0.001. **D**TMB level between low and high-risk groups. **E–F** The quantification analysis of different subtypes in two risk groups of GSE66229 cohort

To comprehend the immune-related molecular characteristics of the different risk groups, maftools were employed and it was demonstrated that the mutation rates of genes in the low-risk group were higher than those in the high-risk group (Fig. 7A, B). Tumor mutational burden (TMB) level displayed in Fig. 7D showed that low-risk group had higher TMB level, in line with the above results. Since higher TMB could predict a better response to immunotherapy [28, 29], these results suggested that the patients in low-risk group might be good candidates for immunotherapy.

TME analyses by ssGSEA and CIBERSIRT methods showed that the low-risk groups were mainly infiltrated by activated CD4^+^ T cells, in line with the results of KGRC1. Therefore, CD4^+^ T cells might be the target for immunotherapy in low-risk *KDM*-related group of GC patients (Fig. 7C). Furthermore, we found that regulatory T cells (Tregs) were mainly enriched in high-risk group. As previously reported, Tregs were main population of immune-suppressive cells [30, 31]. Thus, high-risk group with worse prognosis might exhibit an ineffective response to immunotherapy.

**Fig. 8:**
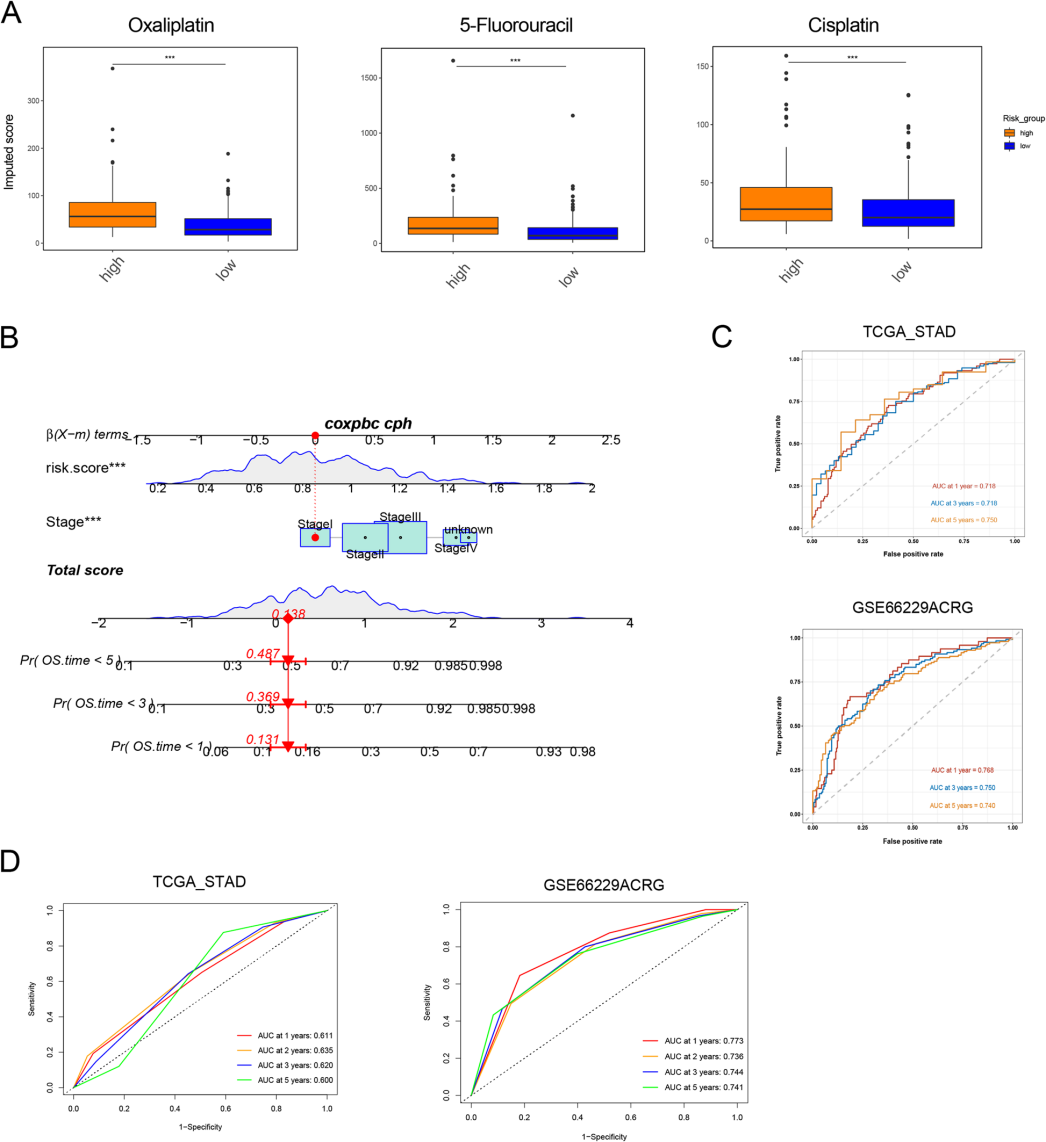
Constructing a nomogram based on *KDM*_score. **A** Drug score between two risk groups. **B-D** ROC curve shows AUC values of nomogram in predicting 1-, 2-, 3-, and 5-year survival of patients in training dataset and validation cohort

As the validation cohort (AGRC cohort) contained epithelial and mesenchymal phenotypes (EP and MP), a proportion analysis was conducted and it was discovered that the high-risk group of the AGRC cohort had a greater number of patients with MP (Fig. 7E), which is known to be associated with a poorer prognosis. Furthermore, it was observed that the high-risk group of the AGRC cohort had more patients with the epithelial-mesenchymal transition (EMT) phenotype (Fig. 7F), while the low-risk group had more patients with the microsatellite instability (MSI) phenotype. As previously reported, EMT was a negative factor [32], while MSI was a positive factor of immunotherapy [33]. So, patients in low-risk group indeed could respond effectively to immunotherapy. Drug susceptibility in the low- and high-risk groups was also evaluated. Interestingly, it was discovered that patients in the high KDM_score group had a higher imputed score for oxaliplatin, 5-fluorouracil, and cisplatin, implying that patients with a high KDM_score may not respond effectively to these drugs (Fig. 8A). Overall, the KDM_score that was constructed may be utilized to predict the response of gastric cancer patients to both immunotherapy and chemotherapy.

### Constructing a nomogram based on *KDM*_score

A nomogram was constructed using the KDM_score and TNM stages to predict overall survival (OS) in the TCGA-STAD cohort. The AUC for survival at 1, 3, and 5 years exhibited high accuracy in the training set (TCGA-STAD) and validation set (GSE66229-ACRG) (Fig. 8B, C). In training set, AUC values at 1-, 3-, and 5-year were 0.718, 0.718, and 0.750, respectively. In validation set, AUC values at 1-, 3-, and 5-year were 0.768, 0.750, and 0.740. By compared with AUC values of TNM stage systems, we found that, in training set, AUC values of nomogram at 1-, 3-, 5-year were higher than that of disease stages (Fig. 8D). In validation set, AUC values of nomogram at 3-year were higher than that of disease stages (Fig. 8D). Finally, the calibration plots of the nomogram shown in Fig. S5A, B suggested that our nomogram has a good prediction ability.

## Discussion

*KDMs* are enzymes that catalyze site-specific demethylation of lysine residues on histones [34], thereby regulating the methylation of H3K4, H3K9, H3K27, or H3K36. Through this process, KDM genes play crucial roles in regulating transcription, chromatin architecture, and cellular differentiation, which can affect the expression of tumor suppressor genes or oncogenes [6]. *KDM* genes have been shown to regulate TME infiltration. For example, *KDM6B ablation has been found to promote CD4*^+^ *T cell differentiation into Th2 and Th17 subsets in the small intestine and colon *[35]*. To identify potential therapeutic targets for personalized treatment of GC, it is crucial to comprehensively understand the correlation between KDM genes and TME characteristics in gastric cancer.*

This study identified three distinct molecular subtypes of gastric cancer related to the KDM gene. The TCGA-STAD cohort was classified into three phenotypes: KGRC1-3. The study also demonstrated that these subtypes exhibit unique characteristics in the tumor microenvironment (TME). Specifically, KGRC1 showed an activation of CD4 + T cells. Talking of the TME traits, CD4^+^T cells helps CD8^+^T cells differentiate into cytotoxic CD8^+^T cells through conventional dendritic cells’ cytokines, such as *IL-12*, *IL-15* and type I interferon [36]. Subsequent ESTIMATE analyses also confirmed the high infiltration level of TME cells in KGRC1, suggesting immune cells in KGRC1 could indeed be the target cells for immunotherapy. Thus, patients in KGRC1 featuring higher activated CD4^+^ T cells might display a better response to immunotherapy. We have introduced for the first time a classification of KDM genes in GC and found that this classification can highlight the immune infiltration status of gastric cancer patients characterized by different KDM genes, providing a new research perspective for the clinical use of immunotherapy in GC patients.

This study also screened the expression of KDMs in tumor and normal samples, and identified KDM5C as highly expressed in gastric cancer. KDM5C was selected for examination of its functional phenotype in GC tumorigenesis, and the results demonstrated that its overexpression could enhance tumor cell metastatic potential and promote xenograft tumor growth. Previous studies indicated that *KDM5C* predicted higher tumor immunogenicity and inflamed anti-tumor immunity alterations [37]. There need to be more studies of *KDM5C* in regulation of tumor microenvironment in gastric cancer. To demonstrate the clinical significance of *KDM* genes, a stable and concise prognostic *KDM*_score was built. Based on the *KDM*_score, patients could be stratified into high-risk and low-risk group showing different prognosis, clinicopathological features and immune infiltration. Furthermore, combining *KDM*_score and tumor stage, we established a comprehensive nomogram to improve the predictivity and accuracy of *KDM*_score. Furthermore, we confirmed the ability of *KDM*_score in immunotherapy and chemotherapy prediction, which we believed that *KDM*_score could be applied in clinical practice to predict patients’ response to immunotherapy and chemotherapy.

To sum up, mutations and expression alterations of *KDM* genes were firstly analyzed in gastric cancer. Then, we figured out KGRC and *KDM*_score. Their correlation with immune infiltration and clinical features in TME were screened out in our research. Nevertheless, our work also has certain shortcomings. This study is mainly based on public database. Further validation in multi-center dataset may better prove our findings.

## Supplementary Information

**Additional file 1.** Supplementary cell line and figure 2B

**Additional file 2. **Supplementary figures

**Additional file 3.** Supplementary table 1

**Additional file 4.** Supplementary table 2

## Data Availability

All data in this study can be obtained from the Gene-Expression Omnibus (GEO; https://www.ncbi.nlm.nih.gov/geo/), the GDC portal (https://portal.gdc.cancer.gov/) and the UCSC Xena (https://xenabrowser.net/datapages/).

## Abbreviations

GC: Gastric cancer
CNV: Copy number variation
GEO: Gene-Expression Omnibus
GSVA: Gene set variation analysis
KGRC: *KDM* Genes-related clusters
TCGA: The Cancer Genome Atlas
TME: Tumor microenvironment
STAD: Stomach Adenocarcinoma
EP: Epithelial phenotype
MP: Mesenchymal phenotype
TMB: Tumor mutational burden
Tregs: Regulatory T cells
