# Supplementary figures and images for: Comprehensive transcriptomic analyses identify KDM genes-related subtypes with different TME infiltrates in gastric cancer
Source: BMC Cancer. 2023 May 18;23:454. doi: 10.1186/s12885-023-10923-1 (PMC10197475; doi:10.1186/s12885-023-10923-1)

Figure 2B

KDM5C

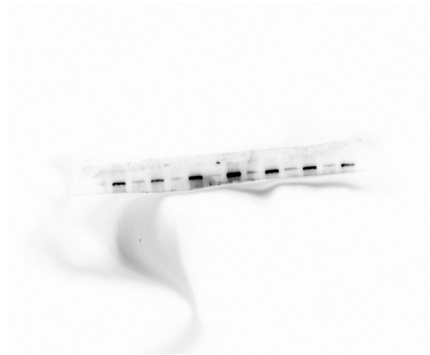

GAPDH

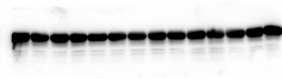

supplementary cell line 2A

KDM5C

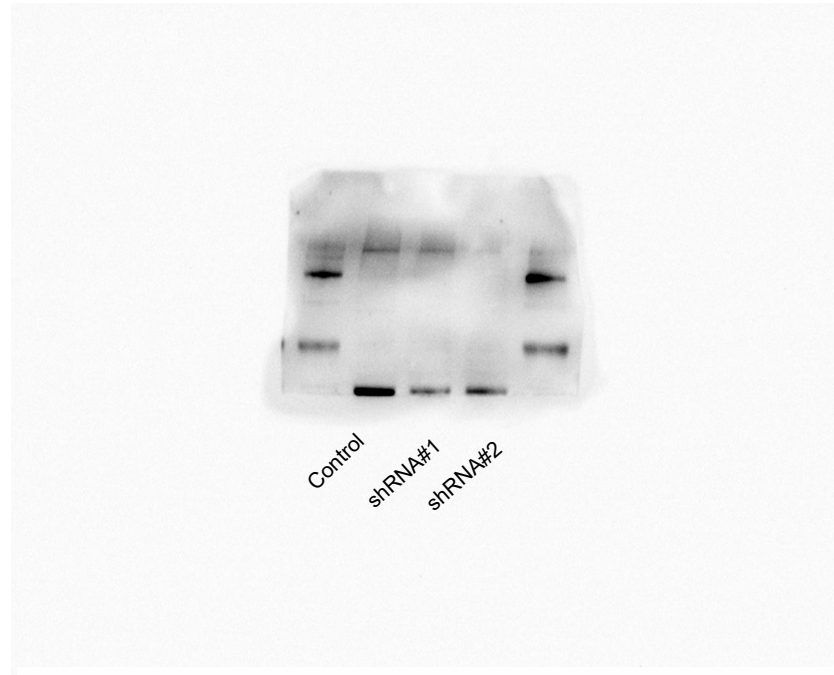

GAPDH

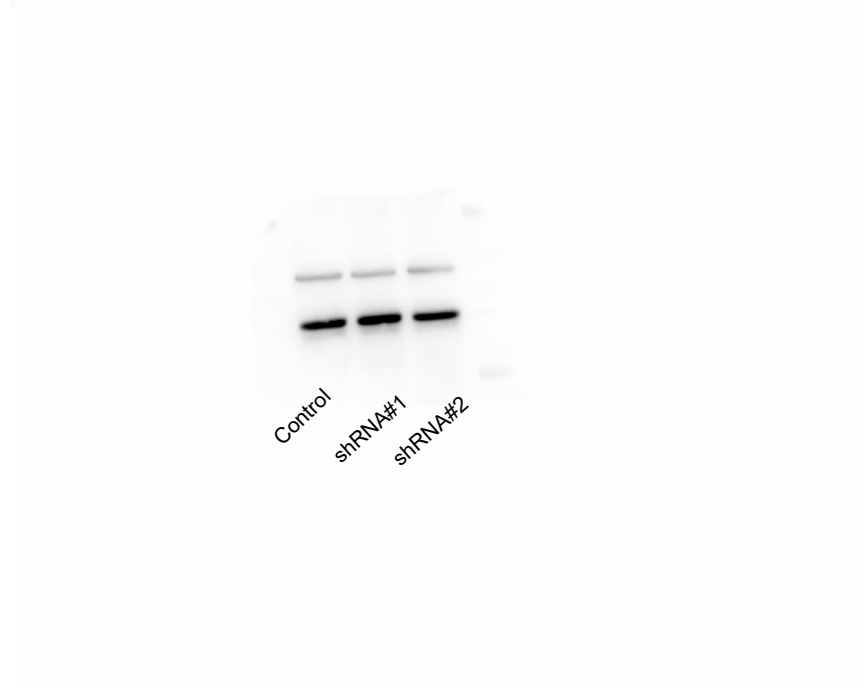

KDM5C

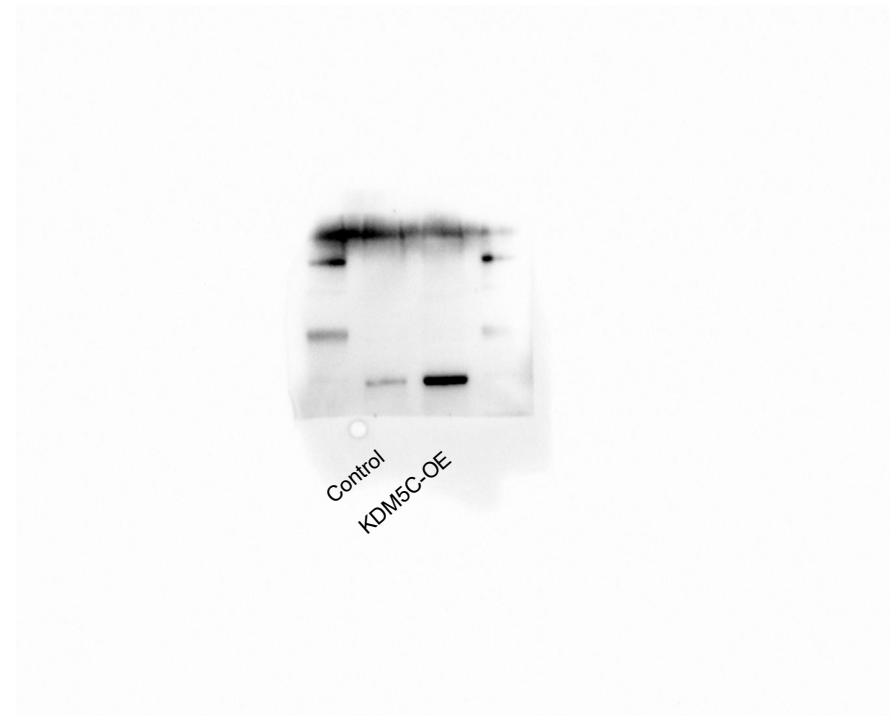

GAPDH

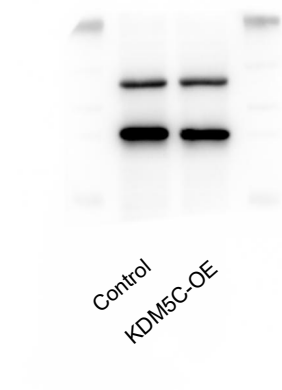

Supplement: Supplementary file 1 — Additional file 1. Supplementary cell line and figure 2B [file 12885_2023_10923_MOESM1_ESM.pdf]
