# Supplementary material for: Comprehensive transcriptomic analyses identify KDM genes-related subtypes with different TME infiltrates in gastric cancer
Source: BMC Cancer. 2023 May 18;23:454. doi: 10.1186/s12885-023-10923-1 (PMC10197475; doi:10.1186/s12885-023-10923-1)
Supplement: Supplementary file 2 — Additional file 2. Supplementary figures [file 12885_2023_10923_MOESM2_ESM.doc]

**Supplemental file 1**

**Comprehensive transcriptomic analyses identify KDM genes-related subtypes with different TME infiltrates in gastric cancer**

**Running title: KDM genes-related gene expression profiling in gastric cancer**

**Authors:**

Haichao Zhang^1†^, Haoran Wang^2†^, Li Ye^3†^, Ji Che^3†^, Ruijia Zhang^3^, Suyun Bao^4^, Wenqin Luo^3^*, Cheng Yu^5^*, Wei Wang^6^*

1 Department of Osteoporosis and Bone Disease, Huadong Hospital affiliated to Fudan University, Research Section of Geriatric Metabolic Bone Disease, Shanghai Geriatric Institute, Shanghai China.

2 Department of Anesthesiology, Zhongshan Hospital, Fudan University, Shanghai, 200032.

3 Department of Oncology, Shanghai Medical College, Fudan University, Shanghai, China.

4 Department of Anesthesiology, The Affiliated Suqian Hospital of Xuzhou Medical University, Suqian 223800, Jiangsu Province, China.

5 Gastrointestinal Surgery, Changshu No.2 People’s Hospital, No.18, Taishan Road, Changshu, Jiangsu, 215500, China.

6 Department of Clinical Laboratory, Lianshui County People's Hospital, Kangda College of Nanjing Medical University, Huai'an, 223400 P.R. China.

†Haichao Zhang, Haoran Wang, Li Ye and Ji Che contributed equally to this work.

*Correspondence equally: wangwei_shzu@163.com (Wei Wang); yucheng10202022@163.com (Cheng Yu); 15627283894@163.com (Wenqin Luo)

Corresponding author:

Wei Wang, M.D.

Department of Clinical Laboratory, Lianshui County People's Hospital, Kangda College of Nanjing Medical University, Huai'an, 223400 P.R. China.

Email: wangwei_shzu@163.com


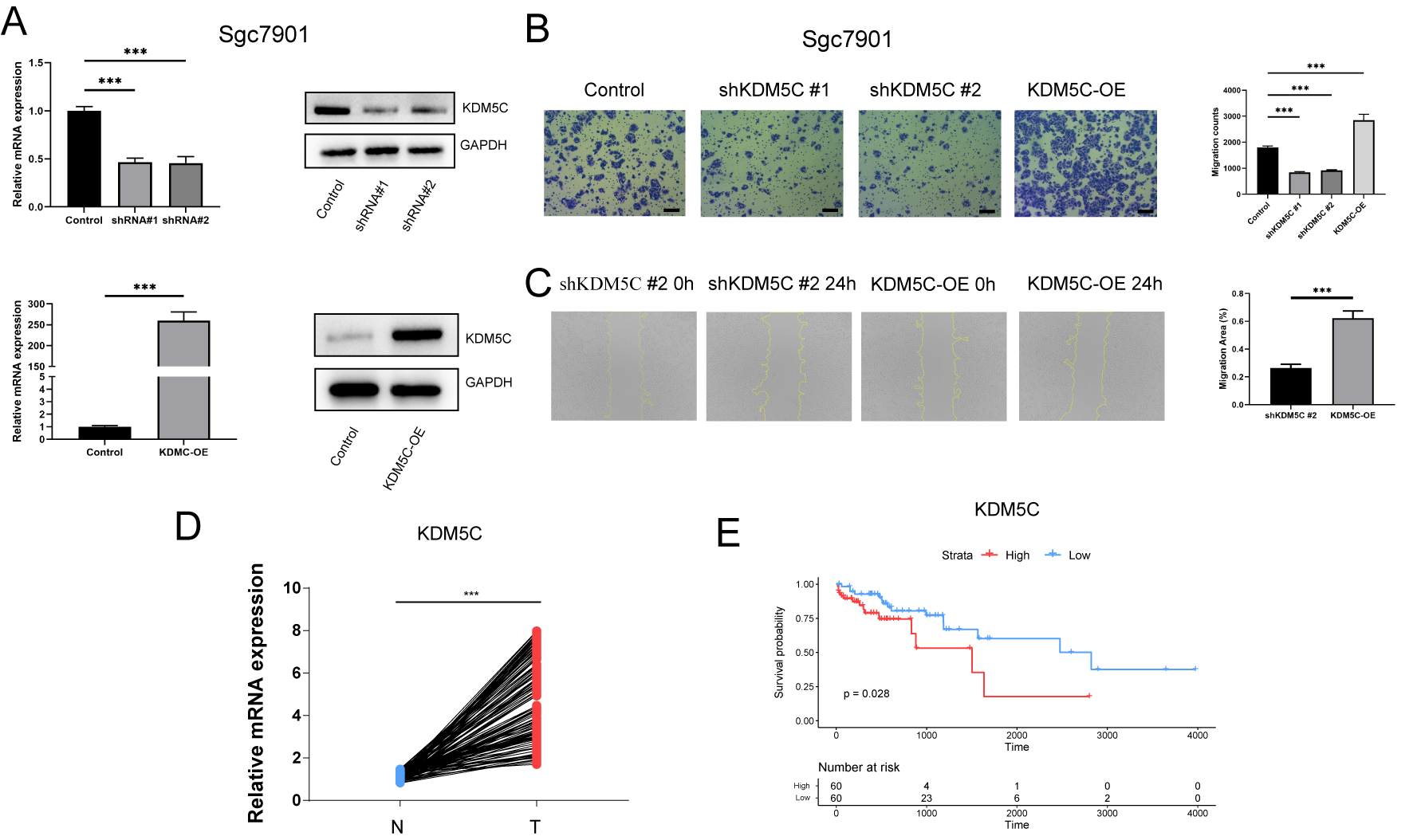


**Fig. S1. Effects of KDM5C on functional study in Sgc7901 cell line and K-M survival analyses based on RNA expression of KDM5C in gastric cancer.**

**(A)** Western blotting and qPCR analyses of overexpression and known-down of KDM5C in Sgc7901 cell line. Statistical difference is identified by t-test, * indicating P value < 0.05; ** indicating P value <0.01; *** indicating P value <0.001. **(B)** Transwell assays of Sgc7901 cell line. Statistical difference is identified by t-test, * indicating P value < 0.05; ** indicating P value <0.01; *** indicating P value <0.001. **(C)** Cell wound scratch assays of Sgc7901 cell line. Statistical difference is identified by t-test, * indicating P value < 0.05; ** indicating P value <0.01; *** indicating P value <0.001. **(D)** PCR assay in 120 pairs of tissues and obtained relative mRNA abundance of KDM5C. **(E)** Kaplan-Meier survival analyses based on RNA expression of KDM5C in our cohort. Statistical difference is identified by t-test, * indicating P value < 0.05; ** indicating P value <0.01; *** indicating P value <0.001.


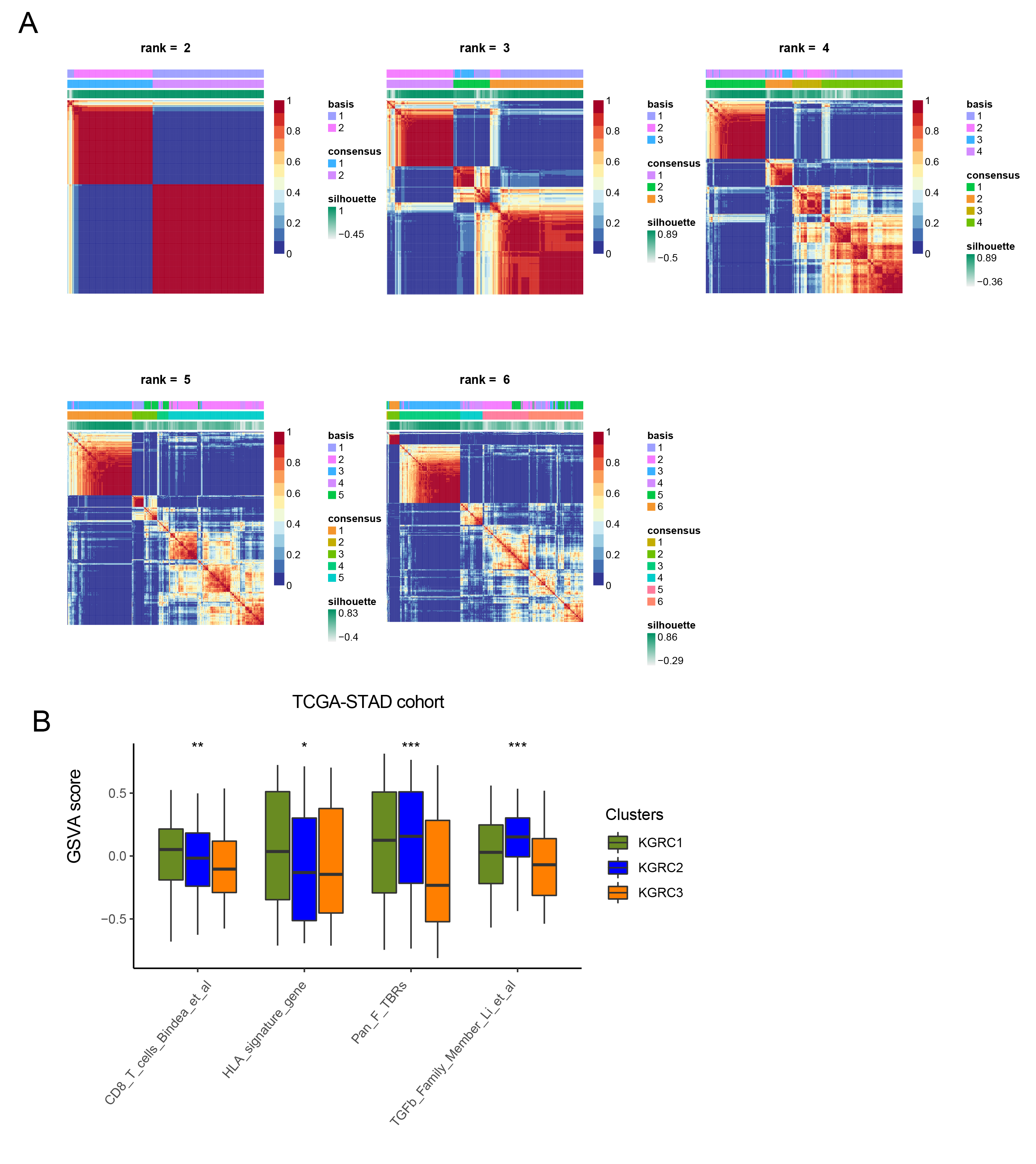


**Fig. S2. Identification of KDM subtypes in GC, related to Figure 1.**

**(A)** Heatmap representation of consensus clustering for necroptosis-related genes in TCGA cohort with cluster numbers from 2 to 6. **(B)** Boxplot shows the GSVA score between three KGRCs.

**
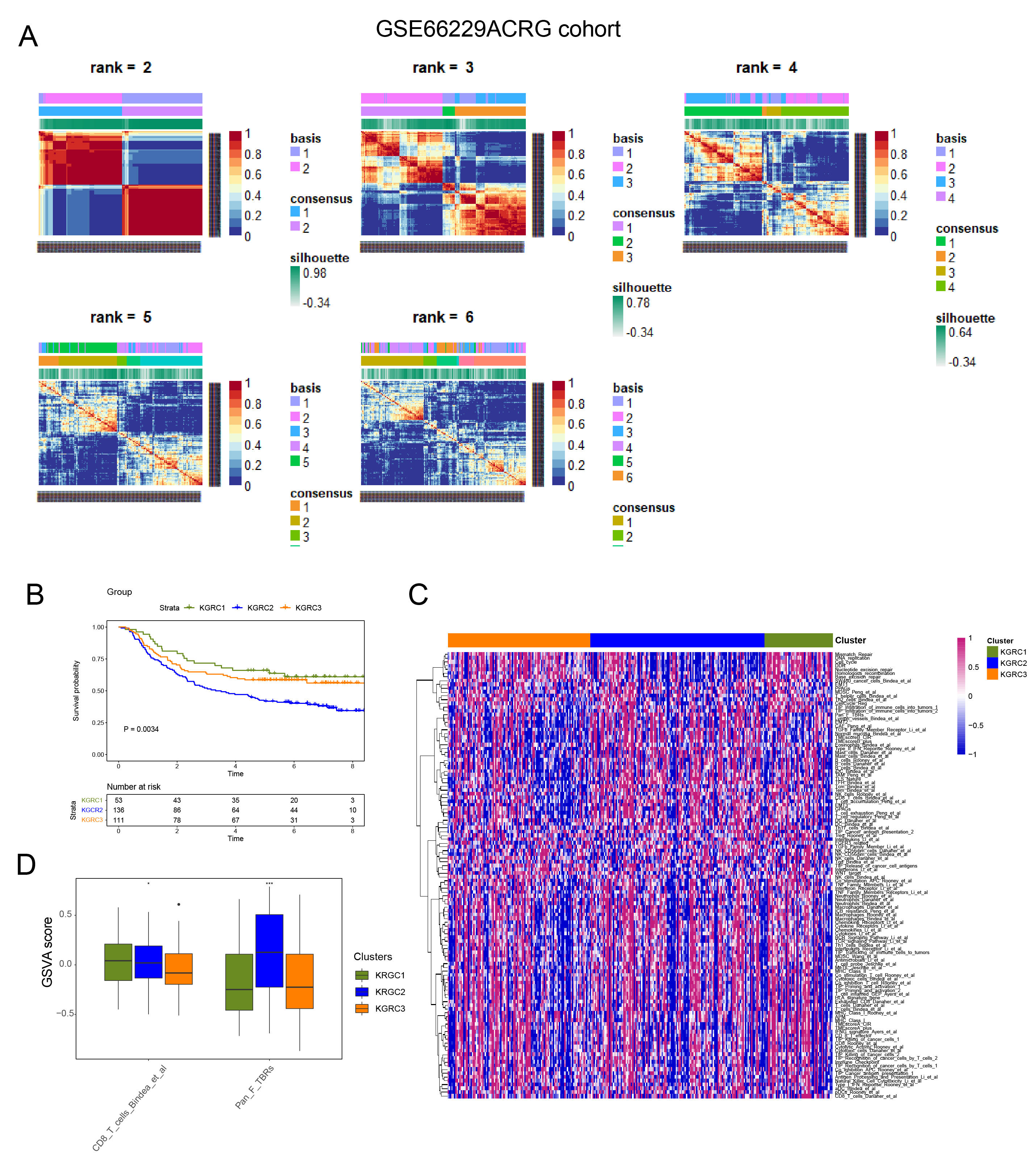
**

**Fig. S3. Identification of KDM subtypes in GC, related to Figure 1.**

**(A)** Heatmap representation of consensus clustering for necroptosis-related genes in GSE66229 cohort with cluster numbers from 2 to 6. **(B)** Kaplan-Meier survival plot for overall survival in GSE66229 cohort is based on 3 KGRCs sorted by NMF algorithm. P value was calculated by LogRank test. **(C)** The enrichment difference of biological pathways in three KGRCs was displayed in heatmap. **(D)** Boxplot shows the GSVA score between three KGRCs.


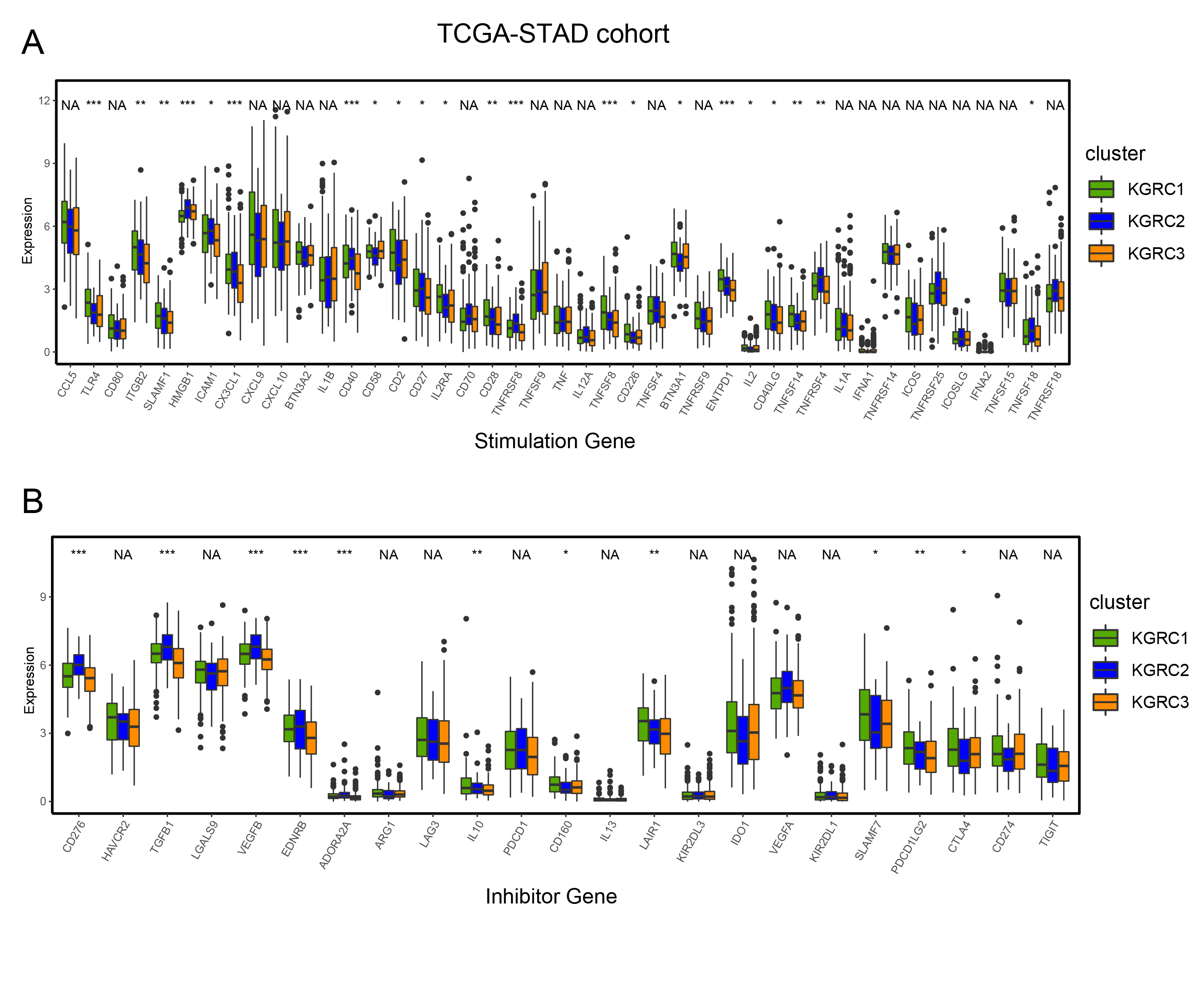
**Fig. S4. Tumor microenvironment infiltration of KGRCs., related to Figure 4**

**(A-B)** Boxplot reflects 23 immune cells infiltration in three KGRCs using ssGSEA algorithm in GSE66229 cohort. Statistical difference is identified by Kruskal-Wallis H test, * indicating P value < 0.05; ** indicating P value <0.01; *** indicating P value <0.001.


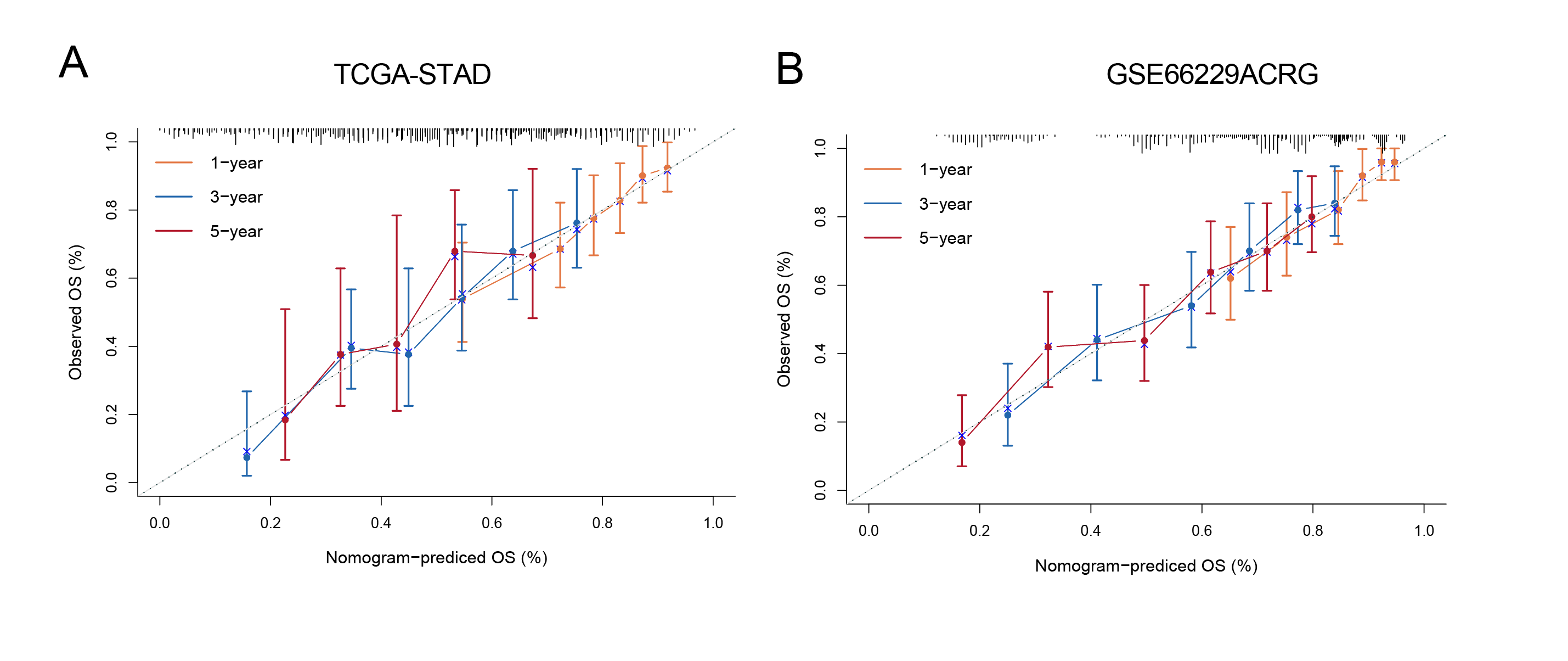


**Fig. S5. Nomogram of GSE66229 cohort.**

**(A-B)** Calibration curves of the nomogram for predicting of 1-, 3-, and 5-year survival rate in the training and testing cohort.

**B. Supplementary Tables**

**Supplementary Table S1.** The description of patients of TCGA-STAD cohort, including clinical characteristics and consensus clusters.

*Please see separated Excel spreadsheet file.*

**Supplementary Table S2.** Lasso_coef used for calculating KDM_score.

*Please see separated Excel spreadsheet file.*
